# Prolonged Mechanical Ventilation in Critically Ill Patients: Six-Month Mortality, Care Pathways, and Quality of Life

**DOI:** 10.1016/j.chest.2025.01.018

**Published:** 2025-01-27

**Authors:** Nicolas Paul, Elena Ribet Buse, Julius J. Grunow, Stefan J. Schaller, Claudia D. Spies, Andreas Edel, Björn Weiss

**Affiliations:** a Charité—Universtätsmedizin Berlin, corporate member of Freie Universität Berlin and Humboldt-Universität zu Berlin, Department of Anesthesiology and Intensive Care Medicine (CCM/CVK), Berlin, Germany; b Medical University of Vienna, Department of Anaesthesia, Intensive Care Medicine and Pain Medicine, Clinical Division of General Anaesthesia and Intensive Care Medicine, Vienna, Austria

**Keywords:** chronic critical illness, critical illness, post ICU care, post intensive care syndrome, prolonged mechanical ventilation, quality of life, weaning

## Abstract

**Background:**

There is limited knowledge about long-term mortality, care pathways, and health-related quality of life (HrQoL) among patients in the ICU receiving prolonged mechanical ventilation (PMV).

**Research Question:**

What are the long-term mortality, care pathways, and HrQoL of patients receiving invasive PMV, stratified by weaning success?

**Study Design and Methods:**

We conducted a secondary analysis of patients from the cluster-randomized controlled Enhanced Recovery After Intensive Care trial who were treated in 2 ICU clusters and received invasive PMV (≥ 21 days via endotracheal tube/tracheostomy or ≥ 4 days via tracheostomy). Data on weaning success, mortality, care place transitions, readmissions, and HrQoL were collected for 6 months after ICU discharge.

**Results:**

Of 90 patients receiving PMV in the ICU, 46% (41 of 90 patients) died (21 patients in the ICU and 20 patients within 6 months after ICU discharge). Of 69 patients discharged alive, 25% (17 of 69 patients) could not be weaned, whereas 75% (52 of 69 patients) were successfully weaned within 6 months. Patients experienced a median of 3 (Q1, Q3: 2, 5) care place transitions within 6 months, with more care place transitions among successfully weaned patients (median, 4 [Q1, Q3: 2, 5] vs 2 [1, 3], *P* = .004). The readmission rate among all patients was 46% within 6 months. One-half of the successfully weaned patients transitioned home, whereas unsuccessfully weaned patients mostly transitioned from weaning centers to nursing homes or died. Unsuccessfully weaned patients had fewer quality-adjusted life days within 6 months than successfully weaned patients (median, 0 [Q1, Q3: 0, 32.6] vs 73.1 [23.2, 135], *P* = .002).

**Interpretation:**

Our results show that three-quarters of patients receiving PMV who were discharged alive were weaned, but their HrQoL was reduced. The decision to proceed with PMV should weigh in patient preferences for living with HrQoL limitations and patients’ likelihood of weaning.

**Clinical Trial Registration:**

ClinicalTrials.gov; No.: NCT03671447; URL: www.clinicaltrials.gov

**Take-Home Points**

**Study Question:** What are long-term mortality, care pathways, and health-related quality of life (HrQoL) of patients receiving prolonged invasive mechanical ventilation?

**Results:** Despite high rates of weaning success, these patients showed a high mortality rate and low HrQoL in the 6 months after ICU discharge. Unsuccessfully weaned patients had worse long-term care pathways than successfully weaned patients.

**Interpretation:** The chance of weaning success, diminished HrQoL, and high mortality need to be considered when deciding to proceed with prolonged mechanical ventilation.

Approximately 1 in 4 patients admitted to an ICU receives mechanical ventilation.^1^ Typically, mechanical ventilation can be discontinued after 3 to 4 days.^1^ However, approximately 1 in 20 mechanically ventilated patients requires prolonged mechanical ventilation (PMV).^2^ Although there are heterogenous definitions for PMV, it is most often defined as ventilation for ≥ 21 consecutive days^3^^,^^4^ or ≥ 4 days with tracheostomy placement.^3^ The total number of patients requiring PMV has been steadily increasing over the last decades.^5^^,^^6^

About 29% of patients receiving PMV die in the hospital, and 50% are successfully weaned from mechanical ventilation.^7^ If weaning fails, patients receiving PMV are transferred to lower-grade care institutions such as long-term acute care hospitals or rehabilitation.^2^, ^3^, ^4^ In the year after discharge, patients receiving PMV have a higher mortality rate, use more health care resources, and incur 3 times more health care costs than ICU patients ventilated for ≤ 21 days.^2^ The 1-year direct health care costs per patient receiving PMV amount to $306,135.^8^ Compared with comfort care in the ICU, PMV was associated with costs of $82,411 per quality-adjusted life year gained.^9^ In addition to high costs, studies indicate that patients receiving PMV have a diminished long-term health-related quality of life (HrQoL) and low levels of functional independence,^8^^,^^10^, ^11^, ^12^ despite improvements in the months after discharge.^13^^,^^14^

Considering the high long-term mortality and the poor outcomes of patients receiving PMV, the decision in the ICU to proceed with PMV should be based on a realistic understanding of long-term mortality, care pathways (ie, the pattern of care place transitions), readmissions, and the chance of returning home, and HrQoL. Although essential for well-informed treatment decisions, to our knowledge, the longitudinal care pathways of patients receiving PMV after ICU treatment have only been explored in 1 previous single-center study in the US health care system,^8^ with limited comparability to European health care systems. Considering the excessive costs of patients receiving PMV, understanding their care pathways may also guide health policymakers optimizing care for these patients.

Consequently, this secondary analysis of a cluster-randomized controlled trial aims to investigate the mortality, care pathways based on the pattern and number of care place transitions as well as hospital readmissions, and HrQoL among patients receiving PMV in 3 German ICUs, stratified by weaning success.

## Study Design and Methods

### Study Design, Ethics, Population, and Setting

This is a secondary analysis from the multicenter, stepped-wedge cluster-randomized controlled Enhanced Recovery After Intensive Care (ERIC) trial.^15^^,^^16^ ERIC assessed the impact of a complex telemedicine intervention on the quality of ICU care and was approved by the Ethics Committee of Charité on January 26, 2018 (EA1/006/18), as well as the Ethics Committee of Brandenburg Medical School on October 30, 2018 (Z-01-20180828). Between September 4, 2018, and March 31, 2020, patients were enrolled in 10 ICU clusters in the metropolitan area of Berlin, Germany. Inclusion criteria were as follows: expected ICU length of stay ≥ 24 hours; age ≥ 18 years; and coverage by a German statutory health insurance. Patients or legal representatives gave consent to study participation.

For this analysis, we included patients who received invasive mechanical ventilation for ≥ 21 days via an endotracheal tube/tracheostoma or ≥ 4 days via a tracheostoma and were treated in 2 academic ICU clusters (3 ICUs) out of 10 ICU clusters of the trial. Detailed data on care place transitions and weaning were available for patients from these clusters. We compared baseline characteristics of our study population with patients who met the ventilation inclusion criteria but were enrolled in other clusters. We only considered invasive ventilation and did not analyze noninvasive ventilation during and after ICU treatment. There was no standardized determination of weaning failure across the ICUs and no minimum time before discharging a patient from the ICU on a ventilator. We included patients enrolled in the control and intervention phases of the trial. Because the trial’s telemedicine intervention may have impacted weaning-related outcomes, we compared baseline and outcome variables of patients from ERIC’s control and intervention phases.

### Post-ICU Follow-Ups

Follow-up examinations were scheduled 3 and 6 months after ICU discharge. Patients were contacted via phone or email. Follow-ups were conducted by trained study personnel using a post-intensive care syndrome measurement instrument set to assess patients’ disability, HrQoL, cognition, mental health, and mobility (e-Table 1).^17^ They took place at the study center or as home visits. In rare instances where home or study center visits proved logistically impractical, supplementary follow-up procedures were implemented via telephone or mail. Information regarding patients’ care situation was collected from general practitioners.

### Outcomes, Data Collection, and Variable Definitions

The outcomes of interest for this analysis were mortality, the pattern and number of care place transitions, and hospital readmissions within 3 and 6 months after ICU discharge, as well as HrQoL at follow-ups, stratified by weaning success.

We used data collected during patients’ ICU stay, including demographics, severity of illness (Simplified Acute Physiology Score II [SAPS II] and Sequential Organ Failure Assessment [SOFA] scores), ventilation status in the ICU, ICU and hospital length of stay, and discharge disposition. From follow-up examinations, patients’ general practitioners, and patients’ electronic medical records, we collected data on HrQoL using the EuroQol-5 Dimensions-5 Levels (EQ-5D-5L),^18^ the duration of invasive mechanical ventilation after discharge, the date of successful weaning, and care place transitions. The mortality for up to 6 months after ICU discharge was collected from the municipal personal records database.

As recommended for patients receiving PMV,^4^ successful weaning was defined as not requiring invasive mechanical ventilation for ≥ 7 days. When the exact weaning date was unavailable but could be narrowed down to < 2 months, we used the midpoint of that period. Any change in care locations, including transfers between different wards or hospitals, was considered a transition. Outpatient hospital appointments and deaths were not counted as transitions. If a patient was transferred to a facility with the primary goal of weaning from invasive ventilation, we considered that facility a weaning center, regardless of whether it was formally designated as an ICU, weaning center, or rehabilitation facility. Definitions of care locations are provided in e-Table 2.

EQ-5D-5L index values were derived using the German value set.^19^ HrQoL was categorized as *good*, *fair*, *poor*, or *dead*, depending on patients’ EQ-5D-5L index score. Following a previous study,^8^ an EQ-5D-5L index value of 2 SDs below the German population mean for people of the same age and sex was considered a “poor” HrQoL.^20^ “Good” HrQoL was defined as an EQ-5D-5L index score above the German population mean for people of the same age and sex.^20^ Intermediate scores were described as “fair” HrQoL. Quality-adjusted life days were calculated by multiplying the EQ-5D-5L index value at the first follow-up with the days alive within 3 months after discharge and the EQ-5D-5L index value at the second follow-up with the days alive between 3 and 6 months after discharge.^8^ If only 1 EQ-5D-5L index value was available, it was used for both periods.

### Statistical Analysis

We compared patients with successful and unsuccessful weaning within 6 months after ICU discharge. Categorical variables are presented as absolute and relative frequencies and compared with Pearson χ^2^ tests. Continuous variables are presented as median [Q1, Q3] and compared with Mann-Whitney *U* or Kruskal-Wallis tests, as appropriate. The mortality of ICU survivors who were weaned and ICU survivors who were not weaned within 6 months was compared using Kaplan-Meier estimates and log-rank tests. We computed group-specific Sankey diagrams and swimmer plots to visualize care place transitions. Transitions between HrQoL groups were visualized using an alluvial plot. We used Kaplan-Meier estimates to analyze the weaning success in the months after ICU discharge. Univariable logistic regression models were computed to assess the association between successful weaning after 6 months (dependent variable) and the SAPS II at ICU admission, the total time ventilated in the ICU, and the Glasgow Coma Scale at discharge (each as independent variable) because these were considered potential determinants of weaning success. Analyses were carried out in R (version 4.2.2, R Core Team) and RStudio (Posit Software, PBC, Boston, MA) using the tidyverse, eq5d, and networkD3 packages, and in Stata18 SE (StataCorp LLC, College Station, TX). Because of the exploratory nature of the study, we did not adjust for multiple testing. *P* < .05 was considered significant.

## Results

### Study Population and Mortality

Of 1,463 patients enrolled in the ERIC trial, 655 were enrolled in 2 academic ICU clusters. Of those, 90 patients met the ventilation inclusion criteria. Approximately one-fourth (21 of 90 patients) died during their ICU stay. Most patients (77 of 90 patients) were enrolled in the intervention phase of the ERIC trial, with no differences in study-related outcomes between intervention and control phase patients. Patients who met the ventilation inclusion criteria but were enrolled in other ICU clusters of the ERIC trial had lower SAPS II (*P* = .003) and SOFA (*P* < .001) scores at admission, and had fewer days ventilated during their ICU stay (*P* = .002) (Table 1, e-Fig 1, e-Table 3, e-Table 4, e-Table 5).

**Table 1:** Baseline Characteristics of the Study Population and Trajectories During ICU Stay (N = 90)

Variable	Not Weaned From Ventilation (n = 38)	Weaned From Ventilation (n = 52)	All Patients (N = 90)	*P* Value
Sex, male	27 (71)	36 (69)	63 (70)	.852
Age, y	72 [61.3, 80]	66.5 [56.5, 74.3]	69 [57, 78]	.112
ICU admission mode				.18
Emergency room	6 (16)	14 (27)	20 (22)	
Operating room	12 (32)	23 (44)	35 (39)	
Ward	9 (24)	5 (10)	14 (16)	
Other ICU	6 (16)	4 (8)	10 (11)	
External	5 (13)	6 (12)	11 (12)	
ICU admission diagnosis				.325
Respiratory	5 (13)	6 (12)	11 (12)	
Sepsis/infection	6 (16)	11 (21)	17 (19)	
GI	2 (5)	7 (13)	9 (10)	
Cardiovascular	11 (29)	7 (13)	18 (20)	
Trauma	3 (8)	10 (19)	13 (14)	
Neurologic	7 (18)	8 (15)	15 (17)	
Oncologic	3 (8)	3 (6)	6 (7)	
Other	1 (3)	0 (0)	1 (1)	
ICU admission reason	(n = 37)		(n = 89)	.367
Medical	17 (46)	17 (33)	34 (38)	
Surgical (emergency)	12 (32)	24 (46)	36 (40)	
Surgical (elective)	8 (22)	11 (21)	19 (21)	
SAPS II at admission	54.5 [36.3, 70.5]	47 [34, 55]	49.5 [35.3, 61.8]	**.046**
SOFA at admission	10 [8, 13]	9 [6, 11.3]	10 [8, 12]	.101
Ventilation status at admission				.244
Breathing independently	30 (79)	44 (85)	74 (82)	
Transferred intubated from other ICU	6 (16)	8 (15)	14 (16)	
Long-term ventilated before ICU admission	2 (5)	0 (0)	2 (2)	
Enrolled in intervention phase of ERIC trial	33 (87)	44 (85)	77 (86)	.767
ICU LOS, d	31 [20, 46.5]	31 [23, 43.3]	31 [21.3, 45]	.765
Hospital LOS, d	33.5 [20, 46.5]	36.5 [27, 51.3]	35 [22, 48.8]	.149
ICU mortality	21 (55)	0 (0)	21 (23)	**< .001**
Total time ventilated during ICU stay, d	24.5 [14.8, 42.3]	23.5 [14, 38.5]	24 [14, 40]	.621
Time intubated during ICU stay, d	7 [2, 12.8]	10 [5.75, 14]	8.5 [3, 13.8]	.184
Time with tracheostomy during ICU stay, d	20 [5.25, 32.5]	15 [4.5, 28]	17 [5, 28]	.3
Glasgow Coma Scale at discharge among ICU survivors	10 [7, 13] (n = 17)	11 [8, 14] (n = 50)	11 [7.5, 14] (n = 67)	.399

Data are presented as median [Q1, Q3] or No. (%). Significant *P* values (*P* < .05) are highlighted in bold. Groups were compared using the Pearson χ^2^ test or Mann-Whitney *U* test. In case of missing values, n is indicated in parentheses. ERIC = Enhanced Recovery After Intensive Care; LOS = length of stay; SAPS II = Simplified Acute Physiology Score II; SOFA = Sequential Organ Failure Assessment score.

Of 69 patients who were discharged alive from the ICU, 72% (50 of 69 patients) were ventilated at the time of ICU discharge. Among those discharged alive, 25% (17 of 69 patients) could not be weaned from ventilation within 6 months, whereas 75% (52 of 69 patients) were successfully weaned. Another 29% (20 of 69 patients) died in the 6 months after ICU discharge. Among patients who could not be weaned from mechanical ventilation, 65% (11 of 17 patients) died within 6 months after ICU discharge, compared to 17% (9 of 52 patients) of those successfully weaned (*P* < .001) (Table 2, e-Fig 2).

**Table 2:** Ventilation and Mortality-Related Variables Within 6 Months After ICU Discharge for Patients Discharged Alive From the ICU (N = 69)

Variable	ICU Survivors Not Weaned From Ventilation (n = 17)	ICU Survivors Weaned From Ventilation (n = 52)	All ICU Survivors (N = 69)	*P* Value
Discharged from the ICU with ventilation	16 (94) a	34 (65)	50 (72)	.026
Time with invasive mechanical ventilation in 6 months following ICU discharge, d	69 [17, 180]	13 [0, 30.8] (n = 46)	20 [0, 38.5] (n = 63)	**< .001**
3-mo mortality	10 (59)	5 (10)	15 (22)	**< .001**
6-mo mortality	11 (65)	9 (17)	20 (29)	**< .001**
Days alive 6 months after ICU discharge	69 [17, 180]	180 [180, 180]	180 [135, 180]	**< .001**
Time until death in 6 months after discharge, d	29 [13.5, 60.5] (n = 11)	59 [8, 135] (n = 9)	43 [11, 84] (n = 20)	.342
Ventilation status at death^b^	(n = 11)	(n = 9)	(n = 20)	**.003**
Died with invasive mechanical ventilation	8 (73)	0 (0)	8 (40)	
Died without invasive mechanical ventilation	1 (9)	6 (67)	7 (35)	
Unknown	2 (18)	3 (33)	5 (25)	
Location of death^b^	(n = 11)	(n = 9)	(n = 20)	.52
Ward	2 (18)	2 (22)	4 (20)	
At home	0 (0)	1 (11)	1 (5)	
Other ICU	2 (18)	2 (22)	4 (20)	
Rehabilitation	0 (0)	1 (11)	1 (5)	
Nursing home	2 (18)	2 (22)	4 (20)	
Weaning center	3 (27)	0 (0)	3 (15)	
Unknown	2 (18)	1 (11)	3 (15)	
Circumstances of death^b^	(n = 11)	(n = 9)	(n = 20)	.834
Planned withdrawal from ventilation	5 (45)	4 (44)	9 (45)	
Under full support	1 (9)	1 (11)	2 (10)	
At home	0 (0)	1 (11)	1 (5)	
Unknown	3 (27)	2 (22)	5 (25)	
Nursing home	2 (18)	1 (11)	3 (15)	

Data are prestend as median [Q1, Q3] or No. (%). Significant *P* values (*P* < .05) are highlighted in bold. Groups were compared using the Pearson χ^2^ test or Mann-Whitney *U* test. If No. is different than the respective column's group size, it is indicated in parentheses. Of the 90 patients included in the study, 21 died in the ICU, leaving 69 ICU survivors.

a Successful weaning was defined as not requiring invasive mechanical ventilation for at least 7 d. One patient was discharged to the ward with palliative intention without ventilation and died 3 d later.

b Percentages are expressed in relation to the patients who died.

### Care Place Transitions and Readmissions

We observed 188 and 244 care place transitions within 3 and 6 months after ICU discharge, respectively. Patients experienced a median of 2 [Q1, Q3: 2, 3] care place transitions within 3 months and 3 [Q1, Q3: 2, 5] care place transitions within 6 months. Patients successfully weaned experienced significantly more care place transitions (*P* < .001 and *P* = .004 after 3 and 6 months, respectively). More than one-third (36%, 25 of 69 patients) and almost one-half (46%, 32 of 69 patients) of patients were readmitted within 3 and 6 months, respectively (Table 3).

**Table 3:** Care Place Transitions and Readmissions Within 6 Months After ICU Discharge for Patients Discharged Alive From the ICU (N = 69)

Variable	ICU Survivors Not Weaned From Ventilation (n = 17)	ICU Survivors Weaned From Ventilation (n = 52)	All ICU Survivors (N = 69)	*P* Value
Hospital discharge disposition				.632
Ward	3 (18)	13 (25)	16 (23)	
Other ICU	0 (0)	5 (10)	5 (7)	
Rehabilitation	2 (12)	6 (12)	8 (12)	
Nursing home	1 (6)	2 (4)	3 (4)	
Weaning center	11 (65)	26 (50)	37 (54)	
Care place after 6 mo				**.002**
Home	2 (12)	26 (50)	28 (41)	
Rehabilitation facility	0 (0)	3 (6)	3 (4)	
Ward	1 (6)	4 (8)	5 (7)	
Nursing home	2 (12)	10 (19)	12 (17)	
Other ICU	1 (6)	0 (0)	1 (1)	
Dead	11 (65)	9 (17)	20 (29)	
Care place transitions after 3 mo	2 [1, 2]	3 [2, 4]	2 [2, 3]	**.001**
Care place transitions after 3 mo, total	31	157	188	NA
Care place transitions after 6 mo	2 [1, 3]	4 [2, 5]	3 [2, 5]	**.004**
Care place transitions after 6 mo, total	42	202	244	NA
Readmissions after 3 mo	0 [0, 0]	0 [0, 1]	0 [0, 1]	**.023**
Percentage of patients being readmitted at least once within 3 mo	2 (12)	23 (44)	25 (36)	**.016**
Readmissions after 6 mo	0 [0, 1]	1 [0, 1]	0 [0, 1]	.252
Percentage of patients being readmitted at least once within 6 mo	5 (29)	27 (52)	32 (46)	.106

Data are presented as median [Q1, Q3] or No. (%). Significant *P* values (*P* < .05) are highlighted in bold. Groups were compared using the Pearson χ^2^ test or Mann-Whitney *U* test. In case of missing values, n is indicated in parentheses. Of the 90 patients included in the study, 21 died in the ICU, leaving 69 ICU survivors. NA = not applicable.

**Figure 1:**
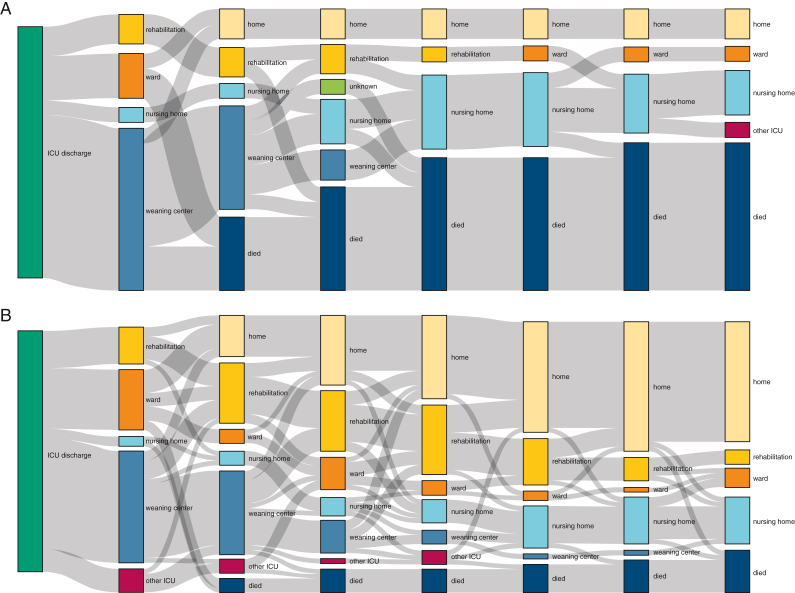
Changes in the place of care within 6 mo after discharge for patients discharged alive from the ICU (N = 69). The Sankey diagrams are grouped by (A) patients with unsuccessful weaning (n = 17) and (B) patients with successful weaning (n = 52). The second bar resembles the discharge disposition. Each following bar represents 1 mo after ICU discharge.

Patients initially discharged to a weaning center transitioned to other care locations in the following months. Even among patients with unsuccessful weaning, very few stayed in weaning centers. They either died or were transferred to nursing homes. Successfully weaned patients gradually transferred from wards and weaning centers to rehabilitation facilities and to home. The share of patients living at home after 6 months was higher among successfully weaned than unsuccessfully weaned patients (50% vs 12%; *P* = .002) (Fig 1, e-Fig 3, e-Fig 4).

**Figure 2:**
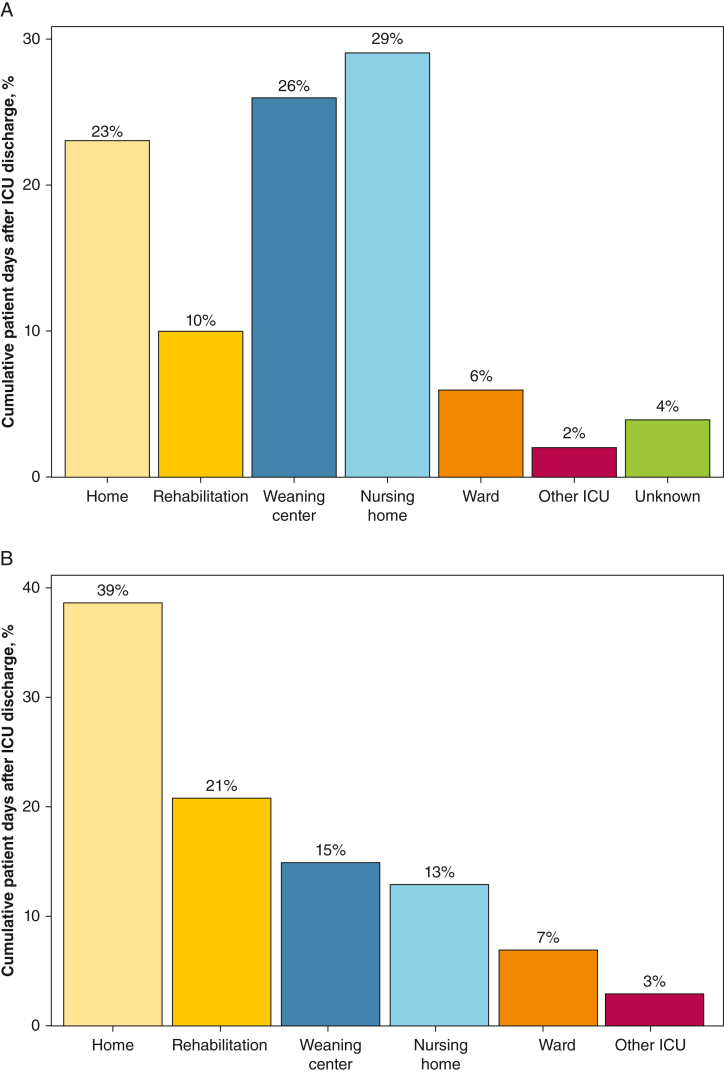
Share of cumulative patient days alive spent at each care place within 6 months after discharge. Bar graphs are grouped by (A) patients with unsuccessful weaning (1,518 patient days for 17 patients) and (B) patients with successful weaning (8,432 patient days for 52 patients).

Of 9,950 patient days spent alive within 6 months after ICU discharge, 37% (n = 3,658) of days were spent at home. Those who were successfully weaned spent more days at home (39% vs 23%) and at rehabilitation facilities (21% vs 10%), but fewer days in weaning centers (15% vs 26%) and nursing homes (13% vs 29%; all *P* < .001) than those who were unsuccessfully weaned (Fig 2, e-Fig 5, e-Table 6).

### Health-Related Quality of Life

At the first follow-up, almost two-thirds of patients had passed away or reported a poor HrQoL grouping (62%, 38 of 61 patients). The HrQoL grouping among patients who could not be weaned was significantly worse compared with those who were successfully weaned (*P* < .001). At the second follow-up, the overall HrQoL groupings showed improvements, with more patients with a good or fair HrQoL grouping (43%, 28 of 65 patients). Unsuccessfully weaned patients had significantly worse EQ-5D-5L index values after 3 and 6 months (*P* = .002 and *P* = .016) and fewer quality-adjusted life days after 3 and 6 months (both *P* = .002) (Table 4, e-Fig 6, e-Table 7).

**Table 4:** Health-Related Quality of Life within 6 Months After ICU Discharge for Patients Discharged Alive From the ICU (N = 69)

Variable	ICU Survivors Not Weaned From Ventilation (n = 17)	ICU Survivors Weaned From Ventilation (n = 52)	All ICU Survivors (N = 69)	*P* Value
3-mo quality of life^a^	(n = 16)	(n = 45)	(n = 61)	**< .001**
Good	0 (0)	4 (9)	4 (7)	
Fair	2 (13)	17 (38)	19 (31)	
Poor	4 (25)	19 (42)	23 (38)	
Dead	10 (63)	5 (11)	15 (25)	
6-mo quality of life^b^	(n = 16)	(n = 49)	(n = 65)	**.002**
Good	1 (6)	5 (10)	6 (9)	
Fair	2 (13)	20 (41)	22 (34)	
Poor	2 (13)	15 (31)	17 (26)	
Dead	11 (69)	9 (18)	20 (31)	
3-mo EQ-5D-5L index value^a^	0 [0, 0.23] (n = 16)	0.41 [0.13, 0.8] (n = 45)	0.28 [0, 0.57] (n = 61)	**.002**
6-mo EQ-5D-5L index value^b^	0 [0, 0.18] (n = 16)	0.37 [0, 0.70] (n = 49)	0.23 [0, 0.67] (n = 65)	**.016**
Quality-adjusted life days 3 mo after discharge^a^	0 [0, 20.5] (n = 16)	36.5 [11.6, 72.1] (n = 45)	25.3 [0, 51.6] (n = 61)	**.002**
Quality-adjusted life days from 3 to 6 mo after discharge^b^	0 [0, 16.3] (n = 16)	33.7 [0, 62.9] (n = 49)	20.3 [0, 60.1] (n = 65)	**.016**
Quality adjusted life days 6 mo after discharge^c^	0 [0, 32.6] (n = 16)	73.1 [23.2, 135] (n = 45)	50 [0, 114] (n = 61)	**.002**

Data are presented as median [Q1, Q3] or No. (%). Significant *P* values (*P* < .05) are highlighted in bold. Groups were compared using the Pearson χ^2^ test or Mann-Whitney *U* test. In case of missing values, n is indicated in parentheses. Of the 90 patients included in the study, 21 died in the ICU, leaving 69 ICU survivors. EQ-5D-5L = EuroQol-5 Dimensions-5 Levels.

a Missing values replaced by values from the second follow-up.

b Missing values replaced by values from the first follow-up. Please refer to the alluvial plot in e-Figure 6.

c Sum of values for the first 3 mo after discharge and from 3 to 6 mo after discharge.

### Determinants of Weaning Success

Most weaning successes occurred within 3 months after discharge. In the univariable logistic regressions, neither the SAPS II at admission (*P* = .362), the total time ventilated during the ICU stay (*P* = .481), nor the Glasgow Coma Scale score at discharge (*P* = .992) were associated with successful weaning after 6 months (Table 5, e-Fig 7).

**Table 5:** Univariable Logistic Regression Models of Determinants of Successful Weaning After 6 Months Among Those Patients Discharged From the ICU With Ventilation

Variable	OR (95% CI)	*P* Value
SAPS II at admission	1.02 (0.98-1.06)	.362
Total time ventilated during ICU stay, d	1.01 (0.98-1.05)	.481
Glasgow Coma Scale score at discharge	1.00 (0.86-1.17)	.992

A number of 50 patients were discharged with ventilation. Of those, 34 patients were successfully weaned from ventilation, and 16 patients could not be weaned after 6 mo. Kaplan-Meier estimates for successful weaning after 6 mo are shown in e-Figure 6. SAPS II = Simplified Acute Physiology Score II.

## Discussion

We analyzed the 6-month mortality; care pathways, defined as the pattern and number of care place transitions and hospital readmissions; and HrQoL stratified by weaning success among 90 patients receiving PMV in 3 ICUs. Six months after ICU discharge, 54% (49 of 90 patients) of the initial cohort were still alive, and their HrQoL was relatively low. However, among 69 patients discharged from the ICU alive, 38% were alive, weaned, and at home after 6 months. Patients with successful weaning experienced more care place transitions, spent more time at home, spent less time in weaning centers and nursing homes, and had a better HrQoL than patients with unsuccessful weaning.

A systematic review reported a pooled in-hospital mortality of 26% (95% CI, 24-28) and a pooled 1-year mortality of 62% (95% CI, 57-67) among patients receiving PMV.^7^ Our estimates of an in-hospital mortality of 23% and a 6-month mortality of 46% were similar. Furthermore, we documented a hospital readmission rate of 46%, identical to a population-based study among 6,662 patients receiving PMV in Canada.^2^

One prospective single-center cohort study from the United States comprehensively explored the care pathways of 126 ICU patients receiving PMV.^8^ Their cohort had a slightly lower severity of illness and a shorter ICU length of stay compared with our study. Comparable to our data, 29% of patients were discharged to long-term acute care facilities, which are step-down units specializing in the care of patients receiving PMV.^4^ After 1 year, 68% (86 of 126 patients) were weaned from ventilation, compared with 75% (52 of 69 patients) of those discharged alive and 58% (52 of 90 patients) of the initial cohort after 6 months in our study. Patients experienced a median of 4 [Q1, Q3: 3, 5] care place transitions in 1 year, compared with 3 [Q1, Q3: 2, 5] within 6 months in our study.^8^ The higher number of transitions is particularly remarkable because intrahospital transfers (eg, from the ICU to the ward) were excluded.^8^ Just as in our analysis, successfully weaned patients had more care place transitions and hospital readmissions than unsuccessfully weaned patients.^8^ More care place transitions may indicate a dynamic care situation and imply that patients advance in their recovery process. For example, successfully weaned patients may be discharged from weaning centers to rehabilitation facilities and home more quickly.

In our study, only 9% of ICU survivors showed a good HrQoL after 6 months. Previous findings on the HrQoL of patients receiving PMV have been heterogeneous. In the cohort study mentioned before, 11% were functioning independently and 19% had a good quality of life after 1 year.^8^ Although low, these numbers are higher than in our cohort after 6 months, which may have been due to the higher age in our cohort. Another study on the 1-year functional status of patients receiving PMV showed that 63% described their health as good to excellent.^10^ On the contrary, another study showed that after 3 years, the HrQoL of patients receiving PMV was worse than that in the general population.^11^ The discrepancies, however, may be explained by the high loss to follow-up rates in these studies.^10^^,^^11^

We retrospectively identified patients with successful and unsuccessful weaning, but we were unable to find predictors of weaning success. Predicting weaning success is particularly important for clinical decision-making, because patients with weaning failure showed profoundly different care place patterns, a lower HrQoL, and a higher mortality in our analysis. Previous studies found that a higher SAPS II, a higher Cumulative Illness Rating Scale score, a higher Rapid Shallow Breathing Index score, a cardiac admission diagnosis, a higher pre-admission Zubrod score, readmissions to the respiratory unit, and respiratory comorbidities decreased the likelihood of weaning success, whereas a higher static compliance, a higher prealbumin level, neurological comorbidities, and failed extubation attempts before admission increased the weaning likelihood.^13^^,^^21^^,^^22^

The fact that 75% (52 of 69 patients) of those discharged alive were weaned and 38% (26 of 69 patients) were alive, off the ventilator, and home after 6 months is encouraging for patients, caregivers, and clinicians. At the same time, our study revealed high mortality rates and a reduced HrQoL, especially among unsuccessfully weaned patients. Hence, the decision whether to proceed with PMV or transition to a palliative care goal is highly dependent on individual patient preferences. Based on patients’ core values and personhood, patients and caregivers need to weigh the likelihood of living with a diminished HrQoL and the likelihood of passing away after withdrawal of ventilator support. The treating physicians need to guide and support in the decision process. However, a previous study has shown that physicians and surrogates have unrealistic expectations of chances of survival, quality of life, and functional independence.^23^ The study also found a discordance in surrogate and physician expectations and a lack of physician-surrogate communication about the patient’s prognosis.^23^ Another study found that patients receiving PMV commonly report pain, severe distress, and sadness.^24^ A lack of knowledge about the prognosis may result in ill-guided treatment decisions that do not align with patient preferences. Our study contributes to understanding the long-term trajectories of patients receiving PMV. Well-informed treatment decisions aligned with patient preferences appear even more vital considering the enormous economic costs of patients receiving PMV,^2^^,^^3^^,^^6^^,^^8^ especially for those with limited survival probability.^9^^,^^12^

Many previous studies recruited patients receiving PMV in weaning centers or long-term acute care facilities.^21^^,^^22^^,^^25^, ^26^, ^27^, ^28^ However, patients admitted to those institutions are already subject to a selection bias. We, on the contrary, provide longitudinal follow-ups of patients from their ICU stay onward. We reduced selection bias by conducting home follow-up visits for patients unable to visit the study center. Our data were collected in a multicenter trial with broad inclusion criteria. Hence, our sample resembles a broad spectrum of medical and surgical ICU patients. Notably, we applied a commonly used definition of PMV.^4^^,^^8^^,^^12^ Previous studies have used different PMV definitions,^3^^,^^4^^,^^29^ which limits the comparability of the results.^12^ Our study also has limitations. Most importantly, because of detailed post-ICU data documentation, we could only consider patients receiving PMV from 2 of 10 clusters, resembling 45% of ERIC’s study population. This resulted in a relatively small sample size. All included ICUs were from academic institutions in the metropolitan area of Berlin, Germany. This limits the external validity of our findings for patients treated in smaller, non-university hospitals and other contexts and health care systems in which post-ICU care for patients requiring PMV may be organized differently. Germany, for example, has a network of specialized weaning centers, whereas in the United States, patients may be transferred to long-term acute care facilities that care for a variety of patients requiring prolonged acute care.^3^ Moreover, we did not assess patients’ pre-ICU HrQoL, although previous studies have shown that the HrQoL of ICU patients before ICU admission is already below population norms,^30^ and that pre-ICU HrQoL is a predictor of post-ICU HrQoL.^31^ Furthermore, patients lost to follow-up may have had worse HrQoL than those with follow-ups, which may have caused an overestimation of the HrQoL in our cohort. Finally, our information about the individual reasons for initiating PMV were limited.

## Interpretation

Our study provides substantial evidence on the previously rarely explored care trajectories of patients receiving PMV. Although patients receiving PMV frequently reported a low HrQoL, three-quarters of those discharged from the ICU alive were weaned from ventilation, and 38% were alive, weaned, and home after 6 months. Successfully weaned patients were less likely to die, were more likely to return home, had more care place transitions, and had more quality-adjusted life days than unsuccessfully weaned patients. Our findings underscore that the decision to proceed with PMV is highly dependent on patient and caregiver values and preferences. Additional research on weaning success predictors may further contribute to well-informed treatment decisions between patients, surrogates, and clinicians.

## Funding/Support

German Innovation Fund (Innovationsfonds) of the Federal Joint Committee (Gemeinsamer Bundesausschuss; G-BA; grant 01NVF16011).

## Financial/Nonfinancial Disclosures

The authors have reported to *CHEST* the following: N. P. reports financial support from Teladoc Health for attending meetings. S. J. S. received grants and nonfinancial support from Reactive Robotics GmbH (Munich, Germany), ASP GmbH (Attendorn, Germany), STIMIT AG (Biel, Switzerland), ESICM (Geneva, Switzerland), grants, personal fees, and nonfinancial support from Fresenius Kabi Deutschland GmbH (Bad Homburg, Germany), grants from the Innovationsfonds of The Federal Joint Committee (G-BA), personal fees from Springer Verlag GmbH (Vienna, Austria) for educational purposes and Advanz Pharma GmbH (Bielefeld, Germany), nonfinancial support from national and international societies (and their congress organizers) in the field of anaesthesiology and intensive care medicine, outside the submitted work. S. J. S. holds stocks in small amounts from Alphabet Inc., Bayer AG, and Siemens AG; these holdings have not affected any decisions regarding his research or this study. C. D. S. reports grants from Gemeinsamer Bundesausschuss/Federal Joint Committee (G-BA)—Innovationsfonds during the conduct of the study. Outside the submitted work, C. D. S. reports grants from Deutsche Forschungsgemeinschaft/German Research Society, grants from Deutsches Zentrum für Luft- und Raumfahrt e. V. (DLR)/German Aerospace Centre, grants from Einstein Stiftung Berlin/Einstein Foundation Berlin, grants from Inneruniversitäre Forschungsförderung/Inner University Grants, grants from Projektträger im DLR/Project Management Agency, grants from Stifterverband/Non-Profit Society Promoting Science and Education, grants from European Society of Anaesthesiology and Intensive Care, grants from the Federal Ministry for Economic Affairs and Climate Action (BMWI), personal fees from Georg Thieme Verlag, grants from Dr. F. Köhler Chemie GmbH, grants from Sintetica GmbH, grants from Gemeinsamer Bundesausschuss/Federal Joint Committee (G-BA)—Innovationsfonds, grants from Max-Planck-Gesellschaft zur Förderung der Wissenschaften e.V., grants from Stifterverband für die deutsche Wissenschaft e.V./Metronic, grants from Philips ElectronicsNederland BV, grants from BMBF—Federal Ministry of Education and Research, grants from BMBF/RKI, grants from Deutsche Forschungsgemeinschaft/German Research Society, and grants from the European Commission/Horizon Europe; in addition, C. D. S. has a patent 15753 627.7 issued in Europe (GER; AT; CH; LI; DE; FR; GB; NL) as Inventor, a US patent PCT/EP 2015/067731 issued as inventor, a patent 3 174 588 issued in Europe (GER; CH; LI; DE; FR; NL) as inventor, an international patent 10 2014 215 211.9 licensed, an international patent 10 2018 114 364.8 licensed, an international patent 10 2018 110 275.5 licensed, an international patent 50 2015 010 534.8 licensed, an international patent 50 2015 010 347.7 licensed, and an international patent 10 2014 215 212.7 licensed; in addition, CDS has been part of the data safety monitoring board/advisory board of Prothor and Takeda Pharmaceutical Company Ltd. C. D. S. has unpaid roles in the AWMF (Association of the Scientific Medical Societies in Germany), the Deutsche Forschungsgemeinschaft (German Research Foundation) review boards, the Deutsche Akademie der Naturforscher Leopoldina e. V.—German National Academy of Sciences—Leopoldina, the Berliner Medizinische Gesellschaft, the European Society of Intensive Care Medicine (ESICM), the European Society of Anaesthesiology and Intensive Care, the Deutsche Gesellschaft für Anästhesiologie und Intensivmedizin (German Society of Anaesthesiology and Intensive Care Medicine, DGAI), the Deutsche Interdisziplinäre Vereinigung für Intensiv- und Notfallmedizin (German Interdisciplinary Association for Intensive Care and Emergency Medicine, DIVI), and the Deutsche Sepsis-Stiftung (German Sepsis Foundation). A.E. reports payments made to his institution from Dräger Medical Deutschland GmbH and Gilead Sciences GmbH. A. E. holds shares under 5000€ from Novaxa, BioNTech, and Bavarian Nordic. B. W. reports consulting fees from Orion Pharma Ltd., payments from Dr. F. Koehler Chemie, and financial support from Teladoc Health for attending meetings, outside the submitted work. B. W. also holds a position as chair of the German Society of Anaesthesiology and Intensive Care Medicine (DGAI). None declared (E. R. B., J. J. G.).

## Supplementary Data

e-Online Data

e-Online Data

e-Online Data
