# Supplementary material for: Prolonged Mechanical Ventilation in Critically Ill Patients: Six-Month Mortality, Care Pathways, and Quality of Life
Source: Chest. 2025 Jan 27;168(1):106–18. doi: 10.1016/j.chest.2025.01.018 (PMC12264340; doi:10.1016/j.chest.2025.01.018)
Supplement: e-Online Data [file mmc1.docx]

**Supplementary material: tables**

**Prolonged mechanical ventilation in critically ill patients: six-month mortality, care pathways, and quality of life**

Nicolas Paul, MD MSc^1^, Elena Ribet Buse, MD^1^, Julius J. Grunow, MD^1^, Stefan J. Schaller, MD MBA^1,2^, Claudia D. Spies, MD^1^, Andreas Edel, MD^1^, Björn Weiss, MD^1,*^

^1^ Charité – Universitätsmedizin Berlin, corporate member of Freie Universität Berlin and Humboldt-Universität zu Berlin, Department of Anesthesiology and Intensive Care Medicine (CCM/CVK), Berlin, Germany

^2^ Medical University of Vienna, Department of Anaesthesia, Intensive Care Medicine and Pain Medicine, Clinical Division of General Anaesthesia and Intensive Care Medicine, Vienna, Austria

* Corresponding author

**Supplemental tables:**

**p. 2 e-Table 1.** Instrument set used at follow-up examinations.

**p. 3 e-Table 2.** Definitions of care locations considered in the analysis.

**p. 4 e-Table 3.** Baseline characteristics of patients in the Enhanced Recovery after Intensive Care trial who met the ventilation criteria and were either treated in the two academic ICU clusters analyzed or in the remaining eight ICU clusters.

**p. 5 e-Table 4.** Baseline characteristics and trajectories during ICU stay for those discharged alive from the ICU (N = 69).

**p. 6 e-Table 5.** Baseline characteristics and outcome variables of study patients enrolled in the control phase and intervention phase of the ERIC trial (N = 90).

**p. 7 e-Table 6.** Cumulative patient days alive spent at each care place within six months after ICU discharge.

**p. 8 e-Table 7.** Characteristics and outcomes of patients by health-related quality of life grouping after six months (N = 86).

**e-Table 1.** Instrument set used at follow-up examinations.

| Instrument | Follow-up 1 (after three months) | Follow-up 2 (after six months) |
| --- | --- | --- |
| *Cognition* |  |  |
| MiniCog | X | X |
| Animal Naming | X | X |
| Repeatable Battery for the Assessment of Neuropsychological Status (RBANS) |  | X |
| Trail Making Test A and B (TMT A/B) |  | X |
| *Mental health* |  |  |
| Patient Health Questionnaire-4 (PHQ-4) | X |  |
| Patient Health Questionnaire-8 (PHQ-8) |  | X |
| Generalized Anxiety Disorder-7 (GAD-7) |  | X |
| Impact of Event Scale-Revised (IES-R) |  | X |
| *Mobility* |  |  |
| Timed Up-and-Go (TUG) | X | X |
| Handgrip Strength (HGS) | X | X |
| 2-Minute Walk Test (2-MWT) |  | X |
| Short Physical Performance Battery (SPPB) |  | X |
| *Health-related quality of life* |  |  |
| EQ-5D-5L | X | X |
| *Disability* |  |  |
| WHO Disability Assessment Schedule 2.0 (WHODAS 2.0) | X | X |
| *Subjective health* |  |  |
| Items on subjective mental and physical health before and after the ICU and specific health concerns | X | X |

The assessments are described in Spies CD, Krampe H, Paul N, et al: Instruments to measure outcomes of post-intensive care syndrome in outpatient care settings – results of an expert consensus and feasibility field test. J Intensive Care Soc 2021; 22(2):159-174. doi:10.1177/1751143720923597. ICU, intensive care unit.

**e-Table 2.** Definitions of care locations considered in the analysis.

| Care location | Definition |
| --- | --- |
| Home | Setting where the patient resides either independently or with family members, without the presence of 24-hour on-site medical or nursing staff. Patients may receive frequent visits from a home care service. Long-term invasive mechanical ventilation may be provided at home. Patient may also relocate to a new home after hospital discharge. |
| Nursing home | Residential facility staffed with medical and/or nursing personnel available around-the-clock. Unlike rehabilitation and weaning facilities, there is no therapeutic objective; the focus is on providing long-term care. Nursing homes may also accommodate patients requiring long-term mechanical ventilation. Weaning from invasive mechanical ventilation is not an objective of a nursing home. |
| Rehabilitation facility | Facility dedicated to the patient’s recovery with the aim of improving his or her health status and of facilitating discharge either to their home or to a nursing home. Weaning from invasive mechanical ventilation is not a primary goal of a rehabilitation facility (although it may or may not happen during a patient’s stay in this care location). |
| Weaning center | Any facility or unit, regardless of its formal designation, where the primary goal of the patient's transfer is weaning from invasive mechanical ventilation. This classification is based on the primary care objective, irrespective of the facility's usual function or labelling. |
| Ward | Section within an acute care hospital where patients are neither continuously monitored nor mechanically ventilated. |
| Intensive care unit | Care location equipped for continuous patient monitoring and capable of providing invasive mechanical ventilation. Critically ill patients receive treatment here. |

**e-Table 3.** Baseline characteristics of patients in the Enhanced Recovery after Intensive Care trial who met the ventilation criteria and were either treated in the two academic ICU clusters analyzed or in the remaining eight ICU clusters.

| **Variable** | **Two included academic ICU clusters (n = 90)** | **Remaining eight ICU clusters (n = 93)** | **All patients (N = 183)** | ***p*** |
| --- | --- | --- | --- | --- |
| Sex, male | 63 (70%) | 64 (69%) (n = 92) | 127 (69%) (n = 183) | 0.949 |
| Age, y | 69 [57, 78] | 65 [54, 74] | 67 [55.5, 75.5] | 0.239 |
| ICU admission mode |  |  |  |  |
| Emergency room | 20 (22%) | 34 (37%) | 54 (30%) | 0.07 |
| Operating room | 35 (39%) | 19 (20%) | 54 (30%) |  |
| Ward | 14 (16%) | 16 (17%) | 30 (16%) |  |
| Other ICU | 10 (11%) | 11 (12%) | 21 (11%) |  |
| External | 11 (12%) | 13 (14%) | 24 (13%) |  |
| ICU admission diagnosis |  | (n = 90) | (n = 180) |  |
| Respiratory | 11 (12%) | 17 (18%) | 28 (15%) | 0.774 |
| Sepsis/infection | 17 (19%) | 22 (24%) | 39 (21%) |  |
| Gastrointestinal | 9 (10%) | 8 (9%) | 17 (9%) |  |
| Cardiovascular | 18 (20%) | 17 (18%) | 35 (19%) |  |
| Trauma | 13 (14%) | 7 (8%) | 20 (11%) |  |
| Neurologic | 15 (17%) | 13 (14%) | 28 (15%) |  |
| Oncologic | 6 (7%) | 5 (5%) | 11 (6%) |  |
| Other | 1 (1%) | 1 (1%) | 2 (1%) |  |
| SAPS II at admission | 49.5 [35.3, 61.8] | 42 [32, 51] | 45 [33, 55] | **0.003** |
| SOFA at admission | 10 [8, 12] | 7 [5, 9] | 8 [6, 11] | **<0.001** |
| ICU LOS, d | 31 [21.3, 45] | 30 [22.8, 41.5] (n = 92) | 30 [22, 43.8] (n = 182) | 0.96 |
| Total time ventilated during ICU stay, d | 24 [14, 40] | 18 [11, 25] | 21 [12, 34] | **0.002** |
| Time intubated during ICU stay, d | 8.5 [3, 13.8] | 6 [3, 9] | 7 [3, 11.5] | **0.022** |
| Time with tracheostoma during ICU stay, d | 17 [5, 28] | 11 [5, 20] | 12 [5, 25] | 0.06 |
| ICU mortality | 21 (23%) | 17 (18%) | 38 (21%) | 0.399 |
| Hospital discharge disposition |  | (n = 92) | (n = 182) |  |
| Ward | 16 (18%) | 34 (37%) | 50 (27%) | **<0.001** |
| Other ICU | 5 (6%) | 17 (18%) | 22 (12%) |  |
| Rehabilitation (including weaning centers) | 45 (50%) | 24 (26%) | 69 (38%) |  |
| Nursing home | 3 (3%) | 0 (0%) | 3 (2%) |  |
| Died at ICU | 21 (23%) | 17 (18%) | 38 (21%) |  |

Median [Q1, Q3] or n (%). Significant *p* values (*p*<0.05) are highlighted in bold. Groups were compared using Pearson Chi^2^ test or Mann-Whitney U test. In case of missing values, n is indicated in parentheses. d = days, ICU = intensive care unit; LOS = length of stay; SAPS II = Simplified Acute Physiology Score II; SOFA = Sequential Organ Failure Assessment score; y = years.

**e-Table 4.** Baseline characteristics and trajectories during ICU stay for those discharged alive from the ICU (N = 69).

| **Variable** | **ICU survivors not weaned from ventilation**  **(n = 17)** | **ICU survivors weaned from ventilation**  **(n = 52)** | **All ICU survivors**  **(N = 69)** | ***p*** |
| --- | --- | --- | --- | --- |
| Sex, male | 11 (65%) | 36 (69%) | 47 (68%) | 0.728 |
| Age, y | 65 [53, 79] | 66.5 [56.5, 74.3] | 66 [55, 76] | 0.994 |
| ICU admission mode |  | | | |
| Emergency room | 3 (18%) | 14 (27%) | 17 (25%) | 0.333 |
| Operating room | 6 (35%) | 23 (44%) | 29 (42%) |  |
| Ward | 3 (18%) | 5 (10%) | 8 (12%) |  |
| Other ICU | 4 (24%) | 4 (8%) | 8 (12%) |  |
| External | 1 (6%) | 6 (12%) | 7 (10%) |  |
| ICU admission diagnosis |  | | | |
| Respiratory | 1 (6%) | 6 (12%) | 7 (10%) | 0.334 |
| Sepsis/infection | 1 (6%) | 11 (21%) | 12 (17%) |  |
| Gastrointestinal | 1 (6%) | 7 (13%) | 8 (12%) |  |
| Cardiovascular | 4 (24%) | 7 (13%) | 11 (16%) |  |
| Trauma | 3 (18%) | 10 (19%) | 13 (19%) |  |
| Neurologic | 5 (29%) | 8 (15%) | 13 (19%) |  |
| Oncologic | 1 (6%) | 3 (6%) | 4 (6%) |  |
| Other | 1 (6%) | 0 (0%) | 1 (1%) |  |
| ICU admission reason |  |  |  |  |
| Medical | 7 (41%) | 17 (33%) | 24 (35%) | 0.724 |
| Surgical (emergency) | 6 (35%) | 24 (46%) | 30 (43%) |  |
| Surgical (elective) | 4 (24%) | 11 (21%) | 15 (22%) |  |
| SAPS II at admission | 48 [26, 58] | 47 [34, 55] | 47 [32, 55] | 0.707 |
| SOFA at admission | 9 [8, 11] | 9 [6, 11.3] | 9 [7, 11] | 0.905 |
| Ventilation status at admission |  | | | |
| Breathing independently | 13 (76%) | 44 (85%) | 57 (83%) | **0.042** |
| Transferred intubated from other ICU | 2 (12%) | 8 (15%) | 10 (14%) |  |
| Long-term ventilated before ICU admission | 2 (12%) | 0 (0%) | 2 (3%) |  |
| ICU LOS, d | 35 [22, 42] | 31 [23, 43.3] | 31 [23, 43] | 0.972 |
| Hospital LOS, d | 35 [22, 42] | 36.5 [27, 51.3] | 36 [26, 49] | 0.296 |
| Duration of invasive mechanical ventilation during ICU stay, d | 25 [22, 39] | 23.5 [14, 38.5] | 24 [14, 39] | 0.754 |
| Time intubated during ICU stay, d | 4 [1, 8] | 10 [5.75, 14] | 8 [3, 13] | **0.028** |
| Time with tracheostoma during ICU stay, d | 21 [8, 36] | 15 [4.5, 28] | 16 [5, 29] | 0.214 |
| Total time ventilated during ICU stay and 6 months after discharge, d | 91 [59, 217] | 37 [18.8, 67.8] (n = 46) | 52 [26.5, 81.5] (n = 63) | **0.002** |

Median [25^th^ percentile, 75^th^ percentile] or n (%). Significant *p* values (*p*<0.05) are highlighted in bold. Groups were compared using Pearson Chi^2^ test or Mann-Whitney U test. In case of missing values, n is indicated in parentheses. d = days; ICU = intensive care unit; LOS = length of stay; SAPS II = Simplified Acute Physiology Score II; SOFA = Sequential Organ Failure Assessment score, y = years.

**e-Table 5.** Baseline characteristics and outcome variables of study patients enrolled in the control phase and intervention phase of the ERIC trial (N = 90).

| **Variable** | **Patients enrolled in control phase**  **(n = 13)** | **Patients enrolled in intervention phase**  **(n = 77)** | **All patients**  **(N = 90)** | ***p*** |
| --- | --- | --- | --- | --- |
| Sex, male | 9 (69%) | 54 (70%) | 63 (70%) | 0.948 |
| Age, y | 69 [65, 74] | 70 [57, 79] | 69 [57, 78] | 0.818 |
| ICU admission mode |  | | | |
| Emergency room | 3 (23%) | 17 (22%) | 20 (22%) | 0.753 |
| Operating room | 3 (23%) | 32 (42%) | 35 (39%) |  |
| Ward | 3 (23%) | 11 (14%) | 14 (16%) |  |
| Other ICU | 2 (15%) | 8 (10%) | 10 (11%) |  |
| External | 2 (15%) | 9 (12%) | 11 (12%) |  |
| ICU admission diagnosis |  | | | |
| Respiratory | 3 (23%) | 8 (10%) | 11 (12%) | 0.593 |
| Sepsis/infection | 2 (15%) | 15 (19%) | 17 (19%) |  |
| Gastrointestinal | 2 (15%) | 7 (9%) | 9 (10%) |  |
| Cardiovascular | 1 (8%) | 17 (22%) | 18 (20%) |  |
| Trauma | 1 (8%) | 12 (16%) | 13 (14%) |  |
| Neurologic | 2 (15%) | 13 (17%) | 15 (17%) |  |
| Oncologic | 2 (15%) | 4 (5%) | 6 (7%) |  |
| Other | 0 (0%) | 1 (1%) | 1 (1%) |  |
| ICU admission reason |  | (n = 76) | (n = 89) |  |
| Medical | 7 (54%) | 27 (36%) | 34 (38%) | 0.453 |
| Surgical (emergency) | 4 (31%) | 32 (42%) | 36 (40%) |  |
| Surgical (elective) | 2 (15%) | 17 (22%) | 19 (21%) |  |
| SAPS II at admission | 48.0 [31.0, 62.0] | 50.0 [37.0, 61.0] | 49.5 [35.3, 61.8] | 0.688 |
| SOFA at admission | 5 [0, 10] | 10 [8, 12] | 10 [8, 12] | **0.023** |
| Ventilation status at admission |  | | | |
| Breathing independently | 11 (85%) | 63 (82%) | 74 (82%) | 0.84 |
| Transferred intubated from other ICU | 2 (15%) | 12 (16%) | 14 (16%) |  |
| Long-term ventilated before ICU admission | 0 (0%) | 2 (3%) | 2 (2%) |  |
| ICU LOS, d | 19 [12, 34] | 32 [24, 45] | 31 [21.3, 45] | **0.016** |
| Hospital LOS, d | 19 [12, 38] | 37 [27, 49] | 35 [22, 48.8] | **0.020** |
| ICU mortality | 3 (23%) | 18 (23%) | 21 (23%) | 0.981 |
| 3-month mortality | 6 (46%) | 30 (39%) | 36 (40%) | 0.624 |
| 6-month mortality | 7 (54%) | 34 (44%) | 41 (46%) | 0.516 |
| Successful weaning in the ICU or within 6 months after ICU discharge | 8 (62%) | 44 (57%) | 52 (58%) | 0.767 |
| Care place transitions after 3 months | 2 [2, 3.75] (n=10) | 3 [2, 3] (n = 59) | 2 [2, 3] (n = 69) | 0.951 |
| Care place transitions after 6 months | 3.5 [2.25, 4] (n = 10) | 3 [2, 5] (n = 59) | 3 [2, 5] (n = 69) | 0.89 |
| Readmissions after 3 months | 0 [0, 0.75] (n=10) | 0 [0, 1] (n=59) | 0 [0, 1] (n = 69) | 0.681 |
| Readmissions after 6 months | 0 [0, 1] (n=10) | 0 [0, 1] (n=59) | 0 [0, 1] (n = 69) | 0.688 |
| 3-month quality of life ^a^ | (n = 11) | (n = 71) | (n = 82) |  |
| Good | 0 (0%) | 4 (6%) | 4 (5%) | 0.317 |
| Fair | 4 (36%) | 15 (21%) | 19 (23%) |  |
| Poor | 1 (9%) | 22 (31%) | 23 (28%) |  |
| Dead | 6 (55%) | 30 (42%) | 36 (44%) |  |
| 6-month quality of life ^b^ | (n = 12) | (n = 74) | (n = 86) |  |
| Good | 1 (8%) | 5 (7%) | 6 (7%) | 0.731 |
| Fair | 3 (25%) | 19 (26%) | 22 (26%) |  |
| Poor | 1 (8%) | 16 (22%) | 17 (20%) |  |
| Dead | 7 (58%) | 34 (46%) | 41 (48%) |  |

Median [25^th^ percentile, 75^th^ percentile] or n (%). Significant *p* values (*p*<0.05) are highlighted in bold. Groups were compared using Pearson Chi^2^ test or Mann-Whitney U test. In case of missing values, n is indicated in parentheses. ^a^ Missing values were replaced by values from the second follow-up. ^b^ Missing values replaced by values from the first follow-up. Please refer to the alluvial plot in **e-Figure 6**. d = days; ICU = intensive care unit; LOS = length of stay; SAPS II = Simplified Acute Physiology Score II; SOFA = Sequential Organ Failure Assessment score; y = years.

**e-Table 6.** Cumulative patient days alive spent at each care place within six months after ICU discharge.

| **Care place** | **ICU survivors not weaned from ventilation (1518 days for 17 patients)** | **ICU survivors weaned from ventilation (8432 days for 52 patients)** | **All ICU survivors (9950 days for 69 patients)** | ***p*** |
| --- | --- | --- | --- | --- |
| Home | 344 (23%) | 3314 (39%) | 3658 (37%) | **<0.001** |
| Rehabilitation | 156 (10%) | 1807 (21%) | 1963 (20%) |  |
| Weaning center | 393 (26%) | 1270 (15%) | 1663 (17%) |  |
| Nursing home | 436 (29%) | 1135 (13%) | 1571 (16%) |  |
| Ward | 96 (6%) | 611 (7%) | 707 (7%) |  |
| Other ICU | 34 (2%) | 295 (3%) | 329 (3%) |  |
| Unknown | 59 (4%) | 0 (0%) | 59 (1%) |  |

Significant *p* values (*p*<0.05) are highlighted in bold. ICU = intensive care unit.

**e-Table 7.** Characteristics and outcomes of patients by health-related quality of life grouping after six months (N = 86).

| **Variable** | **good (n = 6)** | **fair (n = 22)** | **poor (n = 17)** | **dead (n = 41)** | ***p*** |
| --- | --- | --- | --- | --- | --- |
| Age, y | 66 [56, 73] | 63 [48.8, 76.5] | 64 [55, 71] | 74 [67, 80] | **0.01** |
| ICU admission diagnosis | | |  | | |
| Respiratory | 2 (33%) | 1 (5%) | 2 (12%) | 6 (14.6%) | 0.269 |
| Sepsis/infection | 1 (17%) | 5 (23%) | 3 (17.6%) | 7 (17.1%) |  |
| Gastrointestinal | 0 (0%) | 3 (14%) | 4 (23.5%) | 2 (4.9%) |  |
| Cardiovascular | 2 (33%) | 2 (9%) | 1 (5.9%) | 12 (29.3%) |  |
| Trauma | 0 (0%) | 7 (32%) | 3 (17.6%) | 2 (4.9%) |  |
| Neurological | 1 (17%) | 3 (14%) | 3 (17.6%) | 7 (17.1%) |  |
| Oncologic | 0 (0%) | 1 (5%) | 1 (5.9%) | 4 (9.8%) |  |
| Other | 0 (0%) | 0 (0%) | 0 (0%) | 1 (2.4%) |  |
| SAPS II at admission | 38.5 [27.8, 53] | 44.5 [30.3, 53.3] | 47 [37, 54] | 55 [43, 71] | **0.015** |
| Ventilation status at admission | | | | | |
| Breathing independently | 5 (83%) | 17 (77%) | 14 (82%) | 35 (85%) | 0.123 |
| Transferred intubated from other ICU | 1 (17%) | 5 (23%) | 1 (6%) | 6 (15%) |  |
| Long-term ventilated before ICU admission | 0 (0%) | 0 (0%) | 2 (12%) | 0 (0%) |  |
| Total time ventilated during ICU stay and 6 months after discharge, d | 55 [18, 79] (n=5) | 42 [31.5, 72.5] (n=19) | 52 [28, 123] (n=16) | 31 [16, 64] | 0.298 |
| Time intubated during ICU stay, d | 5.5 [1.75, 10.8] | 11 [5.25, 14.8] | 8 [6, 12] | 8 [2, 13] | 0.609 |
| Time with tracheostoma during ICU stay, d | 34.5 [8.25, 40.5] | 15.5 [5, 27] | 18 [5, 25] | 14 [5, 28] | 0.881 |
| ICU LOS, d | 40.5 [34.8, 44.8] | 30.5 [25, 45.3] | 33 [24, 36] | 29 [20, 45] | 0.41 |
| Hospital LOS, d | 46.5 [39.8, 48] | 34.5 [26.8, 52] | 39 [33, 58] | 29 [20, 45] | 0.071 |
| Discharged ventilated | 4 (66.7%) | 18 (86%) (n=21) | 10 (59%) | 14 (34%) | **0.003** |
| Hospital discharge disposition | | |  | | |
| Ward | 3 (50%) | 3 (14%) | 5 (29%) | 5 (12%) | **<0.001** |
| Other ICU | 1 (17%) | 2 (9%) | 2 (12%) | 0 (0%) |  |
| Rehabilitation | 0 (0%) | 2 (9%) | 4 (24%) | 1 (2%) |  |
| Nursing home | 0 (0%) | 0 (0%) | 0 (0%) | 3 (7%) |  |
| Weaning center | 2 (33%) | 15 (68%) | 6 (35%) | 11 (27%) |  |
| Died at ICU | 0 (0%) | 0 (0%) | 0 (0%) | 21 (51%) |  |
| Weaning status after 6 months | | |  | | |
| Not weaned from ventilation | 1 (17%) | 2 (9%) | 2 (12%) | 32 (78%) | **<0.001** |
| Weaned from ventilation | 5 (83%) | 20 (91%) | 15 (88%) | 9 (22%) |  |
| 3-month mortality | 0 (0%) | 0 (0%) | 0 (0%) | 36 (88%) | **<0.001** |
| 3-month health outcome grouping | | |  | | |
| Good | 4 (66.7%) | 0 (0%) | 0 (0%) | 0 (0%) | **<0.001** |
| Fair | 1 (16.7%) | 18 (82%) | 0 (0%) | 0 (0%) |  |
| Poor | 1 (16.7%) | 4 (18%) | 17 (100%) | 1 (2%) |  |
| Dead | 0 (0%) | 0 (0%) | 0 (0%) | 36 (88%) |  |
| Missing | 0 (0%) | 0 (0%) | 0 (0%) | 4 (10%) |  |

Median [Q1, Q3] or n (%). Significant *p* values (*p*<0.05) are highlighted in bold. Groups were compared using Pearson Chi^2^ test or Kruskal-Wallis test. In case of missing values, n is indicated in parentheses. Health-related quality of life grouping was missing for 4 patients. d = days; ICU = intensive care unit; LOS = length of stay; SAPS II = Simplified Acute Physiology Score II; SOFA = Sequential Organ Failure Assessment score; y = years.
