# Supplementary material for: Prolonged Mechanical Ventilation in Critically Ill Patients: Six-Month Mortality, Care Pathways, and Quality of Life
Source: Chest. 2025 Jan 27;168(1):106–18. doi: 10.1016/j.chest.2025.01.018 (PMC12264340; doi:10.1016/j.chest.2025.01.018)
Supplement: e-Online Data [file mmc2.docx]

**Supplementary material: figure captions**

**Prolonged mechanical ventilation in critically ill patients: six-month mortality, care pathways, and quality of life**

Nicolas Paul, MD MSc^1^, Elena Ribet Buse, MD^1^, Julius J. Grunow, MD^1^, Stefan J. Schaller, MD MBA^1,2^, Claudia D. Spies, MD^1^, Andreas Edel, MD^1^, Björn Weiss, MD^1,*^

^1^ Charité – Universitätsmedizin Berlin, corporate member of Freie Universität Berlin and Humboldt-Universität zu Berlin, Department of Anesthesiology and Intensive Care Medicine (CCM/CVK), Berlin, Germany

^2^ Medical University of Vienna, Department of Anaesthesia, Intensive Care Medicine and Pain Medicine, Clinical Division of General Anaesthesia and Intensive Care Medicine, Vienna, Austria

* Corresponding author

**Supplemental figures:**

**e-Figure 1.** Flowchart of patients included in the analysis.

**e-Figure 2.** Kaplan-Meier estimates for ICU survivors with unsuccessful weaning (blue) and successful weaning (red). Of 90 patients included in the study, 21 died in the ICU, leaving 69 patients for analysis. Of those, 17 patients could not be weaned from ventilation, and 52 patients were successfully weaned. Log-rank test revealed significant differences between the groups (*p*<0.001). CI = confidence interval; ICU = intensive care unit.

**e-Figure 3.** Sankey diagram showing the changes in the place of care for the first six months after discharge for patients that were discharged alive from the ICU (N = 69). The second bar resembles the ICU discharge disposition. Each following bar represents one month in time for up to six months after ICU discharge. ICU = intensive care unit.

**e-Figure 4.** Swimmer plot illustrating the trajectories of care for the six months after ICU discharge for all patients discharged alive (N = 69). Of 90 patients included, 21 (23%) died in the ICU, leaving 69 (77%) ICU survivors. ICU = intensive care unit.

**e-Figure 5.** The bar graph shows the share of cumulative patient days alive spent at each care place within six months after ICU discharge for the entire sample (9959 patient days for 69 patients). ICU = intensive care unit.

**e-Figure 6.** Alluvial plot showing the health-related quality of life groups at the first and second follow-ups for all patients (N = 90). The first follow-up was scheduled three months after ICU discharge and the second was scheduled six months after ICU discharge. The health-related quality of life was grouped according to patients’ EQ-5D-5L index value. ICU = intensive care unit.

**e-Figure 7.** Kaplan-Meier estimate for successful weaning of those discharged with mechanical ventilation and with exact weaning date available (N = 45). Of 90 patients included in the study, 21 died in the ICU, leaving 69 patients. Of those, 19 patients were discharged without ventilation, leaving 50 patients alive and ventilated. For 5 patients, the exact weaning date was missing, leaving 45 patients for time-to-event analysis. CI = confidence interval; ICU = intensive care unit.
